# Characterization of Electrochemical Processes in Metal–Organic Batteries by X-ray Raman Spectroscopy

**DOI:** 10.1021/acs.jpcc.1c10622

**Published:** 2022-03-16

**Authors:** Ava Rajh, Iztok Arčon, Klemen Bučar, Matjaž Žitnik, Marko Petric, Alen Vizintin, Jan Bitenc, Urban Košir, Robert Dominko, Hlynur Gretarsson, Martin Sundermann, Matjaž Kavčič

**Affiliations:** † Jožef Stefan Institute, Jamova 39, 1000 Ljubljana, Slovenia; ‡ University of Ljubljana, Faculty of Mathematics and Physics, Jadranska ulica 19, 1000 Ljubljana, Slovenia; § University of Nova Gorica, Vipavska 13, SI-5000 Nova Gorica, Slovenia; ∥ University of Zagreb, Faculty of Geotechnical Engineering, Hallerova aleja 7, 42000 Varaždin, Croatia; ⊥ National Institute of Chemistry, Hajdrihova 19, 1000 Ljubljana, Slovenia; ∇ University of Ljubljana, Faculty of Chemistry and Chemical Technology, Večna pot 113, 1000 Ljubljana, Slovenia; ○ Deutsches Elektronen-Synchrotron DESY, Notkestraße 85, D-22607 Hamburg, Germany; ¶ Max Planck Institute for Chemical Physics of Solids, Nöthnitzer Straße 40, D-01187 Dresden, Germany

## Abstract

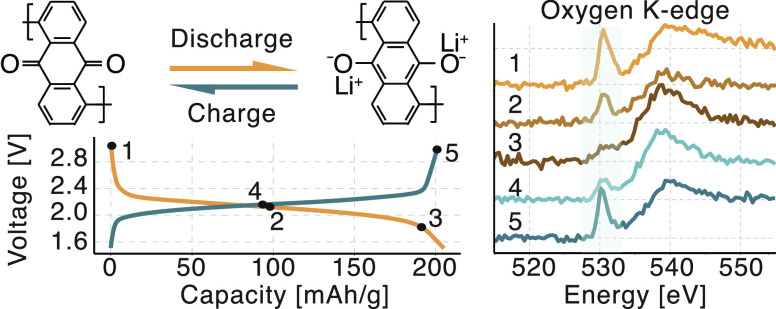

X-ray Raman spectroscopy (XRS) is an emerging spectroscopic technique that utilizes inelastic scattering of hard X-rays to study X-ray absorption edges of low Z elements in bulk material. It was used to identify and quantify the amount of carbonyl bonds in a cathode sample, in order to track the redox reaction inside metal–organic batteries during the charge/discharge cycle. XRS was used to record the oxygen K-edge absorption spectra of organic polymer cathodes from different multivalent metal–organic batteries. The amount of carbonyl bond in each sample was determined by modeling the oxygen K-edge XRS spectra with the linear combination of two reference compounds that mimicked the fully charged and the fully discharged phases of the battery. To interpret experimental XRS spectra, theoretical calculations of oxygen K-edge absorption spectra based on density functional theory were performed. Overall, a good agreement between the amount of carbonyl bond present during different stages of battery cycle, calculated from linear combination of standards, and the amount obtained from electrochemical characterization based on measured capacity was achieved. The electrochemical mechanism in all studied batteries was confirmed to be a reduction of double carbonyl bond and the intermediate anion was identified with the help of theoretical calculations. X-ray Raman spectroscopy of the oxygen K-edge was shown to be a viable characterization technique for accurate tracking of the redox reaction inside metal–organic batteries.

## Introduction

Energy storage technology is a key in the transition from fossil-based energy systems to renewables, due to the intermittent nature of renewable energy sources (wind, sun). Batteries are seen as one of the best energy storage solutions because they are compact and can be used on dispersed locations, which greatly alleviates the need for electrical grid upgrades. Li-ion batteries are, due to their high-energy and high-power density, currently the most mature battery technology and are successfully used in portable electronics, different electromobility applications, and stationary energy storage.^1^ However, with an increasing market demand for Li-ion batteries, this technology is facing concerns regarding its future price, sustainability, and availability of critical raw materials like Co, Ni, and graphite.^2^ These concerns have spurred an increasing interest in the development of post Li-ion battery technologies such as Li–O_2_, Li–S, Na-ion, multivalent batteries, and so forth. Multivalent metal anodes (Mg, Al, and Ca) possess high volumetric and gravimetric capacity while being among the most abundant elements in the Earth’s crust. Unfortunately, almost all inorganic cathodes suffer from poor electrochemical performance, due to difficult insertion of multivalent ions, which prevents the development of practical multivalent batteries. Organic electrodes offer a path to circumvent these limitations since their relatively soft structure can accommodate ions of various charges and sizes. This leads to a high rate performance and versatility with counterion operation.^3,4^ An additional benefit of organic cathodes is the fact that they can be produced from abundant and sustainable materials. Hence, multivalent–organic batteries have emerged as an attractive option for the next generation of batteries.^5^

Organic carbonyl containing polymers with isolated redox-active units, such as members of the quinone family (n-type compounds), are able to undergo reversible reduction and coordinate metal cation. N-type organic compounds paired with metallic anodes enable the design of high-energy organic batteries with consistent charge/discharge voltages,^3^ high capacity, and fast kinetics. Unfortunately, precise mechanisms of the electrochemical reactions inside the organic cathodes are typically not investigated in depth, but often only presumed or investigated by surface sensitive methods like attenuated total reflectance infrared (ATR-IR) or X-ray photoelectron (XPS) spectroscopy.^6,7^

Anthraquinone (AQ) is a well-known redox-active molecule with high theoretical capacity of 257 mAh/g.^8^ AQ displays a well-defined redox voltage plateau at 2.2 V vs Li, which fits its operating voltage window into the operating window of various multivalent electrolytes. This makes AQ an ideal model compound for various multivalent batteries. However, the monomer molecule suffers from high solubility in electrolytes, leading to poor cycling stability. This can be quite effectively mitigated by the preparation of analogous polymers with high molecular weight,^9^ stabilizing the capacity at a cost of decreasing the specific capacity due to the introduction of an electrochemically inactive linker. An example of such polymer is poly(anthraquinonyl sulfide) (PAQS), which contains AQ units connected with sulfur atoms acting as linkers. PAQS showed good reversible electrochemical activity with a varying degree of practical capacity utilization in various multivalent metal anode-organic battery system (Mg, Al, Ca).^10−13^ Additional sulfur atoms in the chain cause a decrease in the specific capacity which can be avoided, if we use a cross-coupling reaction, which directly connects the electroactive groups, as in the case of polyanthraquinone (PAQ) polymer. PAQ polymer displays a good capacity utilization and reversibility and can be cycled for several hundreds of cycles with a small capacity fade.^9^

**Figure 1:**
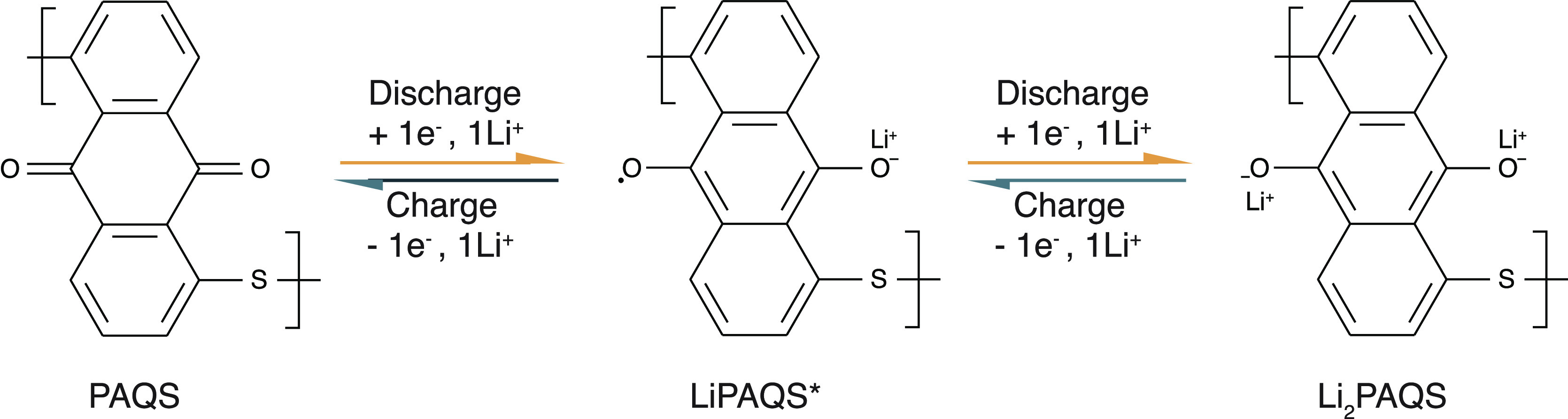
Electrochemical reaction mechanism inside metal–organic battery with poly(anthraquinonyl sulfide) (PAQS) cathode and metallic lithium anode.

Operando measurements based on ATR-IR were recently performed on AQ cathodes with Li^+^ and Mg^2+^ counterions to gain additional information about the reaction mechanism inside the battery.^6,14^ ATR-IR confirmed that the source of electrochemical activity is the reduction of a double carbonyl bond and coordination by metal ion, as presented in Figure 1. Electrochemical mechanisms of PAQ and PAQS were also shown to proceed through a similar redox reaction.^6,12−15^ Although operando ATR-IR is a powerful analytical technique, low penetration depths of a few micrometers probe only the surface of the sample and not the bulk of the material, making it susceptible to degradation of samples and surface contamination. The data obtained from the surface of the electrode can also lead to misleading results if electrochemical reaction is not homogeneous along the whole thickness of the electrode.^6^ Experimental data interpretation of IR spectra is also complicated, since small changes in electronic structure appear as unspecific IR signals, which are often difficult to identify. Moreover, IR characterization also does not allow the quantification of electrochemically active carbonyl bonds within the electrode.^6^

Commonly used spectroscopic characterization technique for studying complex electrochemistry of different battery systems is X-ray absorption spectroscopy (XAS). The method is element selective and sensitive to changes in local electronic structure. Oxygen XAS spectra are in general well studied^16^ and are a useful tool for determining chemical bonding of O atoms. Particularly the transition from 1s orbital to the first unoccupied state in C=O is easily identified by a narrow oscillatory peak at 530 eV. In order to characterize the electrochemical mechanism in PAQ and PAQS organic based batteries, we would need to perform XAS measurements at the oxygen K-edge. Unfortunately, the O K-edge falls in the soft X-ray range (below 1 keV) where the penetration depth of X-rays is limited to around 10–100 nm, again restricting characterization to the surface of the samples. Small penetration depth, combined with required in-vacuum setup, makes soft XAS at the O K-edge unsuitable for bulk studies of oxygen in battery cells. However, this limitation can be circumvented by using hard X-ray spectroscopic technique called X-ray Raman spectroscopy (XRS). XRS is a photon-in/photon-out characterization technique that is based on inelastic scattering of hard X-rays on shell electrons, promoting them to unoccupied states while emitting scattered photons. The signal includes local information about electronic structure and symmetry of target atoms deep into the bulk material. In addition, XRS technique allows the use of both dedicated in situ battery cells or simpler pouch type cells.

Double differential scattering cross section of Raman scattering at low momentum transfer **q**, that is, for small scattering angle between incident and scattered photons, can be written as follows:^17^
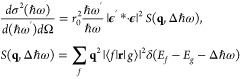
1where **q** = **k** – **k**′ is the momentum transfer delivered to the target by inelastic scattering of incident photon to the scattered photon with respective moments and polarizations (*ℏ***k**, ϵ) and (*ℏ***k**′, ϵ′), *r*_0_ is classical electron radius , Δ*ℏω* = *ℏω* – *ℏω*′ is the energy transferred to the system and the structure factor *S*(*q*, Δ*ℏω*) contains information about excitation of particles from the ground state ⟨*g*| to the final unoccupied states |*f*⟩. The resulting eq 1 represents the measured quantity and is, when energy transfer matches the binding energy of inner shell electrons, directly proportional to the absorption scattering cross section σ_*a*_ at energies around the absorption edge (Δ*ℏω*):^17^
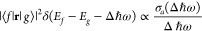
2

By tuning the energy transfer Δ*ℏω* across the particular soft X-ray edge, XRS can be used to measure the scattering signal that is proportional to the soft XAS spectrum of material deep in the sample. Namely, the main advantage in using XRS over more commonly used XAS is that incoming and scattered photons fall in the hard X-ray range (around 10 keV), which enables bulk in-air studies of first row elements with excitation energies in a soft X-ray range.^17^ XRS is, so far, still a relatively new but promising characterization technique that has a cross section of only a few bars. This limits its use to undulator beamlines with dedicated spectrometers based on multiple spherical crystal Bragg analyzers, used to collect scattered photons over a large solid angle with high energy resolution. In this work, we present characterization of four distinct systems consisting of a redox active polymer cathode (PAQ or PAQS) and metallic anode (Li, Mg or Al), using X-ray Raman spectroscopy. All electrodes were cycled under galvanostatic conditions with a C/5 rate. The element-specific electronic structure of the active material in the different metal–organic batteries were studied by looking at the O K-edge of ex situ cathodes during different points of the electrochemical cycle, and comparing it to XRS spectra of standard compounds. This allowed us to quantify the amount of carbonyl bond in a specific sample and provide information about the degree of conversion during the electrochemical reaction. To further interpret the experimental oxygen K-edge XRS spectra, detailed theoretical calculations based on density functional theory (DFT) were performed.

## Experimental Section

### Sample and Electrode Preparation

Polyanthraquinone (PAQ) was synthesized through cross-coupling polymerization of 1,4-dibromoanthraquinone.^18^ Poly(anthraquinoyl sulfide) polymer (PAQS) was synthesized through polycondensation reaction between 1,5-dichloroanthraquinone and Na_2_S. 10% of multiwalled carbon nanotubes were added to the polymerization mixture to improve the capacity utilization of the active material.^12^ Discharged model compounds were prepared by chemical reduction of AQ to 1,10-dihydroxyanthracene, which were subsequently reacted with Li, Mg, and Al organometallic compounds to yield corresponding Li_2_AQ, MgAQ, and (AlCl_2_)-AQ standards.^14^

Electrode composites were prepared by mixing of active material (PAQ, PAQS), Printex XE2 carbon black, and PTFE binder in isopropanol suspension using planetary ball mill Retsch PM100 for 30 min at 300 rpm. In the case of PAQ, the ratio of active material, carbon black, and PTFE was 60:30:10, while in the case of PAQS, the ratio was 80:15:5. A higher amount of active material has been chosen for PAQS electrode to compensate for higher X-ray absorption of PAQS due to presence of sulfur atoms in the polymer. After ball milling, the composite was kneaded in the agate mortar to give the composite with a certain level of plasticity. Obtained composite was subsequently rolled onto glass plate to produce self-standing films of approximately 100 μm thickness from which electrodes with 12 mm diameter were cut. Prepared organic electrodes were cycled in two-electrode setup versus different metal foils (Li, Mg, Al). In the case of Li cells, used electrolyte was 1 M LiTFSI in dimethoxyethane (DME):1,3-dioxolane = 1:1 (volumetric) and voltage range was from 1.5 to 3.5 V vs Li/Li^+^. In the case of Mg cell, 0.6 M Mg(TFSI)_2_-2MgCl_2_ with 50 mM of Bu_2_Mg in DME was used and the voltage range was from 0.5 to 2.5 V vs Mg/Mg^2+^. In the case of Al cells, we used AlCl_3_:EMIMCl = 1.5:1 (molar) electrolyte and the voltage range from 0.4 to 1.8 V vs Al/Al^3+^. In all battery cells we used glassy fiber separators (GF/A Whatman). After disassembly of the cells, electrodes from Li and Mg cells were washed two times in 2 mL of dimethoxyethane (DME) solvent in order to remove the excess electrolyte. Electrodes from Al cell were similarly washed two times in dichloromethane solvent, due to poor solubility of Al electrolyte in DME.

### X-ray Raman Scattering

XRS measurements were carried out at the P01 beamline of PETRA III synchrotron facility in Hamburg. The incoming photon beam, focused to 100 × 100 μm^2^, was directed on the cathode surface at an incident angle between 10° and 15° relative to the surface. The incident energy was set by the Si(311) monochromator and the inelastically scattered photons were analyzed by Raman spectrometer consisting of 12 10 cm diameter Johann-type spherical analysers. A Medipix 2D detector (256 × 256 pixels, 55 × 55 μm^2^) was used to detect scattered photons. In our experiment Si(660) diffraction was used and the emission energy was fixed to 9700 eV. In order to minimize the signal from Compton scattering, measurements were performed at relatively low momentum transfer (2θ ≈ 30°). The transfer energy scans corresponding to oxygen K-edge were achieved by scanning the monochromator over the 10 200–10 270 eV range in 0.2 eV steps. Two consecutive fast scans of 350 steps (0.5 s/step) were performed on a single point on a cathode surface, and then the beam was moved to a new fresh point in order to avoid the radiation damage. The elastic line was measured before the first scan at each point to determine the zero energy transfer and align spectra collected from separate points across the cathode surface. The overall experimental energy resolution was 0.75 eV, as determined from the line width of the elastic scattering signal. Spectra collected from separate points were summed up to get the normalized O K-edge final spectrum. Typically 10–20 separate points were collected for each sample yielding overall acquisition times of 2–3 h to achieve reasonable statistics.

The intrinsic problem with XRS measurements is a very small scattering cross section yielding low count rates. Beside the use of dedicated high efficiency Raman spectrometer, the scattering signal depends on the target cell type. In the case of Li and Mg batteries, the cathodes were enclosed in a Swagelok type cells used in our previous studies on Li–S and Mg–S batteries.^19,20^ A foil, composed of 120 μm polyethylene and 8 μm of Al to prevent outgassing, was used as an entrance/exit window for the beam, separating the cathode from ambient atmosphere. The Swagelok cell allowed for a relatively easy positioning and alignment of the sample relative to the incident beam by the motorized sample holder. However, slight position shifts were observed between the images recorded on the 2D detector when different sample points were brought into the beam. In order to avoid this effect, the Al cathode samples, yielding the weakest signal, were enclosed in a pouch cell, which was evacuated before sealing, yielding a flat window surface resulting in a constant image on the detector despite sample movements in the beam. This stability allowed us to exploit the imaging capabilities of X-ray Raman technique^21^ to optimize the signal to background ratio. This is achieved by isolating the signal from the cathode from other detected signal that originates in X-ray scattering from the entrance window and from the back side of the cell. A more detailed description of this procedure is provided in Supporting Information (SI) S1. In addition, a thinner PE foil (90 μm) was used for the X-ray window to reduce the absorption of incident and scattered photons in the window and enhance the signal intensity. An example of the raw measured signal and the normalized XRS spectrum for one of the Li(PAQS) cathodes is presented in SI S2.

### Theoretical Calculations

Oxygen K-edge XAS spectra for redox active polymers were obtained from first-principle quantum mechanical calculations, using the program package CP2K^22^ based on the density functional theory (DFT). Calculations started with the geometry optimization of molecular structures, where polarized valence triple-ζ (TZV2P)^23^ basis set was used for S, O, C, and H atoms along with Goedecker–Teter–Hutter (GTH)^24^ pseudopotentials. Unknown exchange-correlation potential was substituted with Perdew–Burke–Ernzerhof (PBE),^25^ constructed within general gradient approximation (GGA).^26^ Polymeric structures of PAQ and PAQS were modeled with two monomeric units. Absorption spectra were calculated for each oxygen atom in a molecule. An all electron mixed Gaussian and Plane Wave (GAPW)^27^ approach was implemented.^28^ For O atoms, an all-electron polarization consistent basis (pc-3)^29^ was used.

## Results and Discussion

**Figure 2:**
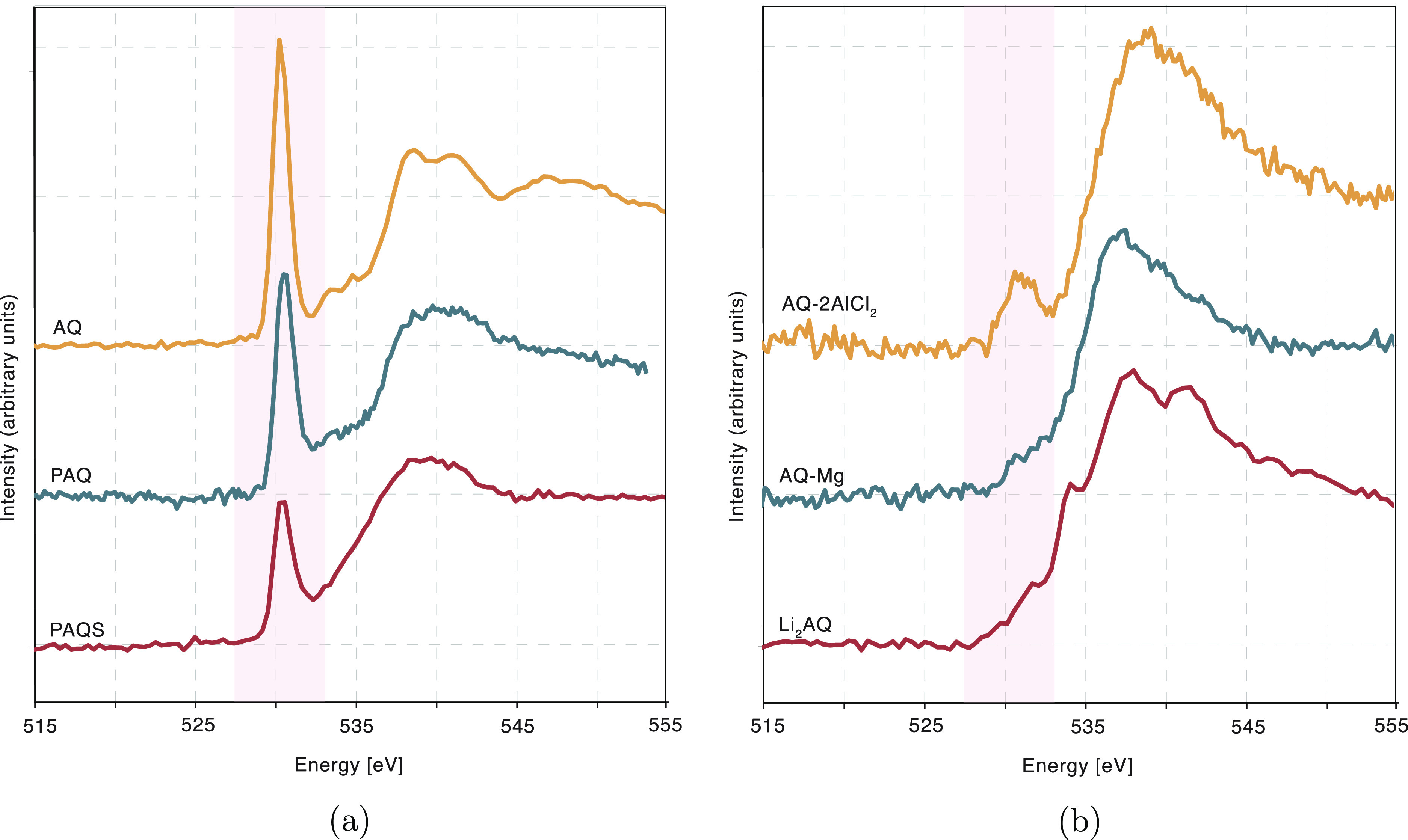
Normalized oxygen K edge XRS spectra of standard compounds (a) representing the initial fully charged state and (b) representing final fully discharged state. In the case of the AQ-2[AlCl_2_] sample, a small carbonyl peak is still present in the measured spectrum. It is attributed to the partial reduction back to the AQ molecule during the reaction post-treatment.

As a starting point for our investigation, O K-edge XRS spectra of several standard compounds were recorded. They were selected to mimic different possible chemical environments of oxygen atoms during the charge/discharge cycle of a battery. Samples of AQ, PAQ, and PAQS were selected to approximate initial state of battery discharge, where all oxygen atoms are bound to carbon with double carbonyl bonds. Reduced anthraquinone salts Li_2_AQ, MgAQ, and AQ-2[AlCl_2_] were selected as closest approximations to the final discharged state of the battery, where all the double carbonyl bonds have been reduced. Comparing the two sets of reference standards in Figure 2, the characteristic resonance at 530 eV is clearly seen in samples representing initial state, and is not present when looking at reference standards for the fully discharged state.

Theoretical XAS spectra of standards were constructed by calculating dipole transition integrals from O 1s ground-state to unoccupied states with O 1s hole. The description of the final states included the core-hole potential on the absorbing atom. From calculated transitions, XAS spectrum was constructed by broadening the stick spectra with a Gaussian profile with a fixed line-width, starting at 0.6 eV and then linearly increasing with excitation energy up to 8 eV to mimic the experimental broadening.^28^ Final spectra were summed over all oxygen atoms in a molecule. All theoretical spectra were shifted by a fixed amount (−0.8 eV in the case of AQ and −0.63 eV for all other cases) to match the experimental excitation energies. The rigid energy shift between the calculated and experimental energy scales originates from the approximation used for the exchange-correlation functional.

**Figure 3:**
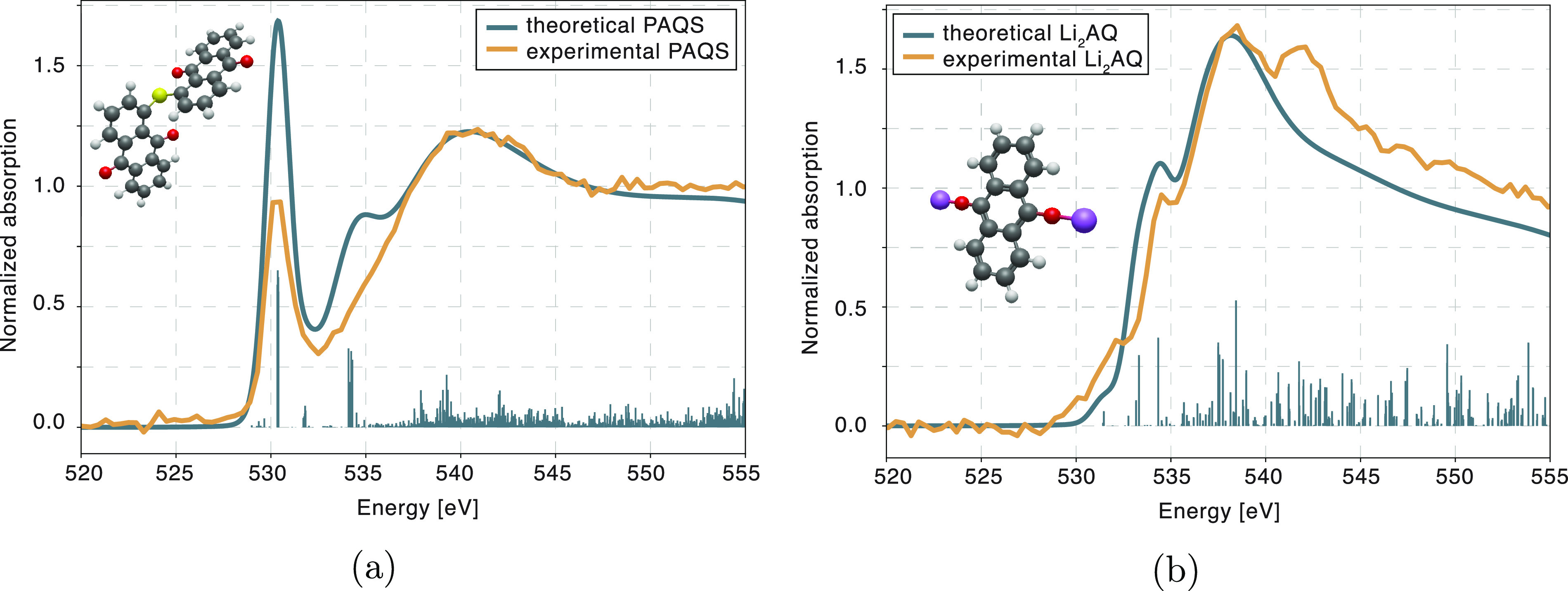
Comparison of the experimental oxygen K-edge XRS spectra and theoretical XAS models obtained by DFT calculations for PAQS and Li_2_AQ molecule.

In general, a good qualitative agreement between theoretical and experimental spectra has been achieved for both monomeric molecules and for approximations of polymeric structures, as can be seen in Figure 3. Spectroscopic signature of the carbonyl bond can be identified in PAQS spectra as a sharp resonance at 530 eV, which is a result of transitions from the 1s to π* orbital. When the bond is reduced, as is the case in Li_2_AQ, the character of π* LUMO state changes to HOMO and the corresponding spectral line disappears. A possible impact of sulfur on the oxygen XAS spectra was further investigated, by calculating theoretical spectra of PAQ and PAQS molecules, presented in SI S4. The spectra were found to be almost identical, confirming that the addition of sulfur atom to the molecule does not affect O K-edge XAS spectrum.

Measured O–K edge spectra of standard samples were used for fitting the ex situ XRS spectra of electrodes stopped at five different points during the charge/discharge cycle. Cathodes from four distinct battery systems were measured: Li-PAQ, Li-PAQS, Mg-PAQS, and Al-PAQS.

**Figure 4:**
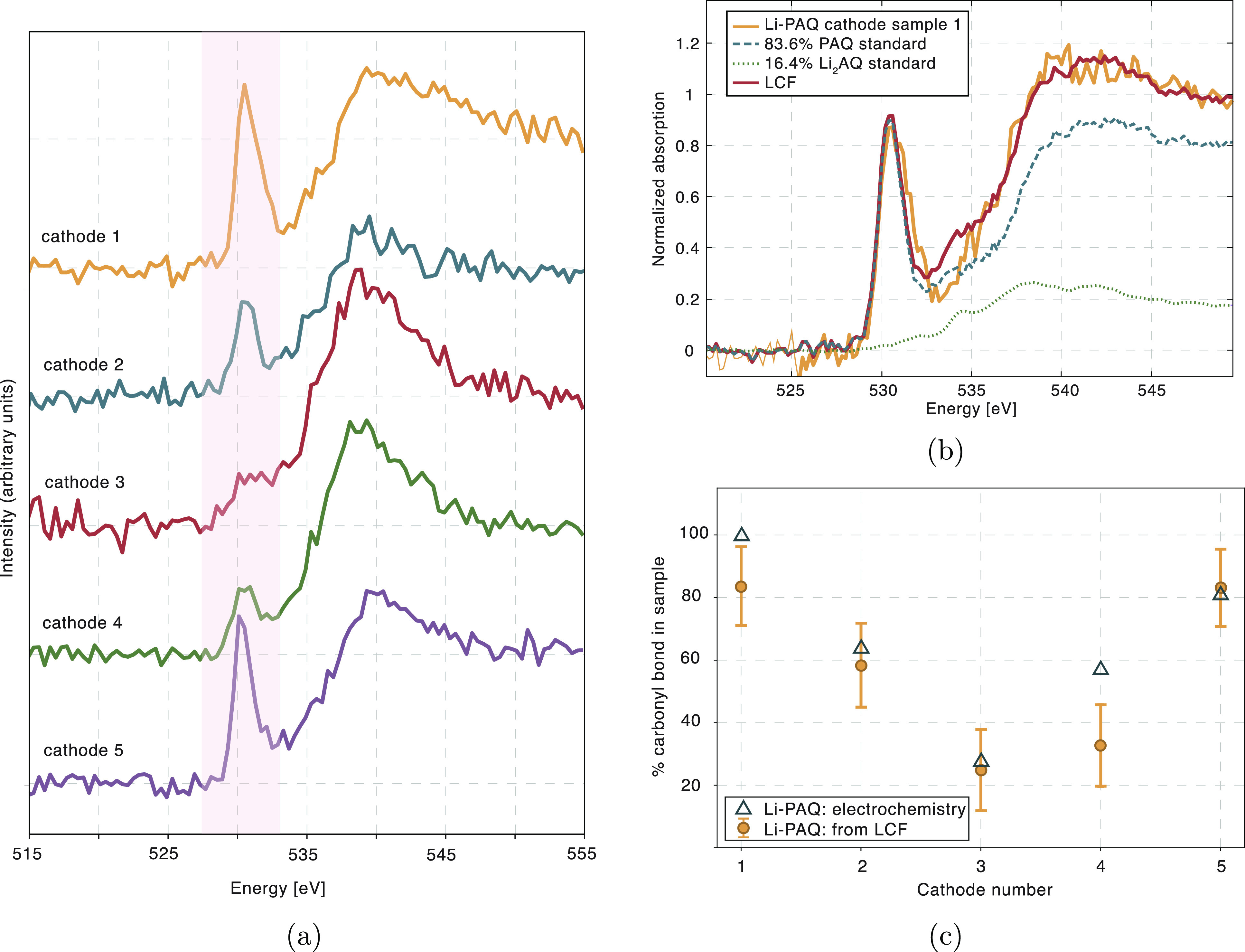
(a) Experimental oxygen K-edge XRS spectra from 5 precycled cathodes measured ex situ from Li-PAQ battery. Cathode numbering corresponds to the consecutive points along the battery cycle presented in Figure S3. Cathode 1 is uncycled pristine cathode, cathode 2 was taken at the halfway point of the discharge, cathode 3 was fully discharged, cathode 4 was half way charged after a complete discharge and cathode 5 was again fully charged. (b) Linear combination fit of two standard compounds (PAQ and Li_2_AQ) to the spectrum from PAQ cathode in the initial state (cathode 1). (c) Comparison of relative amounts of carbonyl bond obtained using LCF analysis of measured spectra and by electrochemical characterization for Li-PAQ battery.

Experimental oxygen XRS spectra of Li-PAQ battery samples measured ex situ are presented in Figure 4 and the corresponding electrochemical discharge and charge curves are shown in SI S3a. PAQ delivered a discharge capacity of around 200 mAh/g, meaning a practical capacity utilization of almost 80%, with an average discharge plateau potential of 2.1 V. The redox potential is well-defined and highly reversible which points to an absence of notable side reactions. Comparison of the measured spectra from ex situ samples in Figure 4a clearly shows the change in relative intensity of the characteristic resonance at 530 eV with time evolution of the cathode. By the end of the discharge phase, the resonance almost vanishes from the spectrum and is recovered back to the initial intensity by the end of the charge. This clearly confirms the evolution of oxygen bonds through the battery cycle. In order to better interpret these results, a quantitative analysis was performed, where individual spectra were modeled using a linear combination of two standard compounds representing beginning and end phases of a battery cycle as presented in Figure 4b. Linear combination fit (LCF) was then performed for measured spectra of all PAQ electrodes with different metallic anodes and relative amounts of double carbonyl bond were determined for individual samples. These values were compared with values obtained from electrochemical characterization based on measured capacities, as seen in Figure 4c. While calculated values from LCF of XRS spectra for Li-PAQ cathode were found to be in good agreement with values gained by electrochemical characterization of samples, it can be seen from the illustration of LCF in Figure 4b, that the spectra of a standard compound PAQ does not accurately describe the spectrum of the first cathode sample. The difference between the two spectra, which were expected to be identical, was attributed to the various additives that make up the finished electrode and possible remainder of the electrolyte even after thorough washing of the electrodes. The difference was considered an estimate for the accuracy of LCF for obtaining the relative amount of carbonyl bonds in a sample.

**Figure 5:**
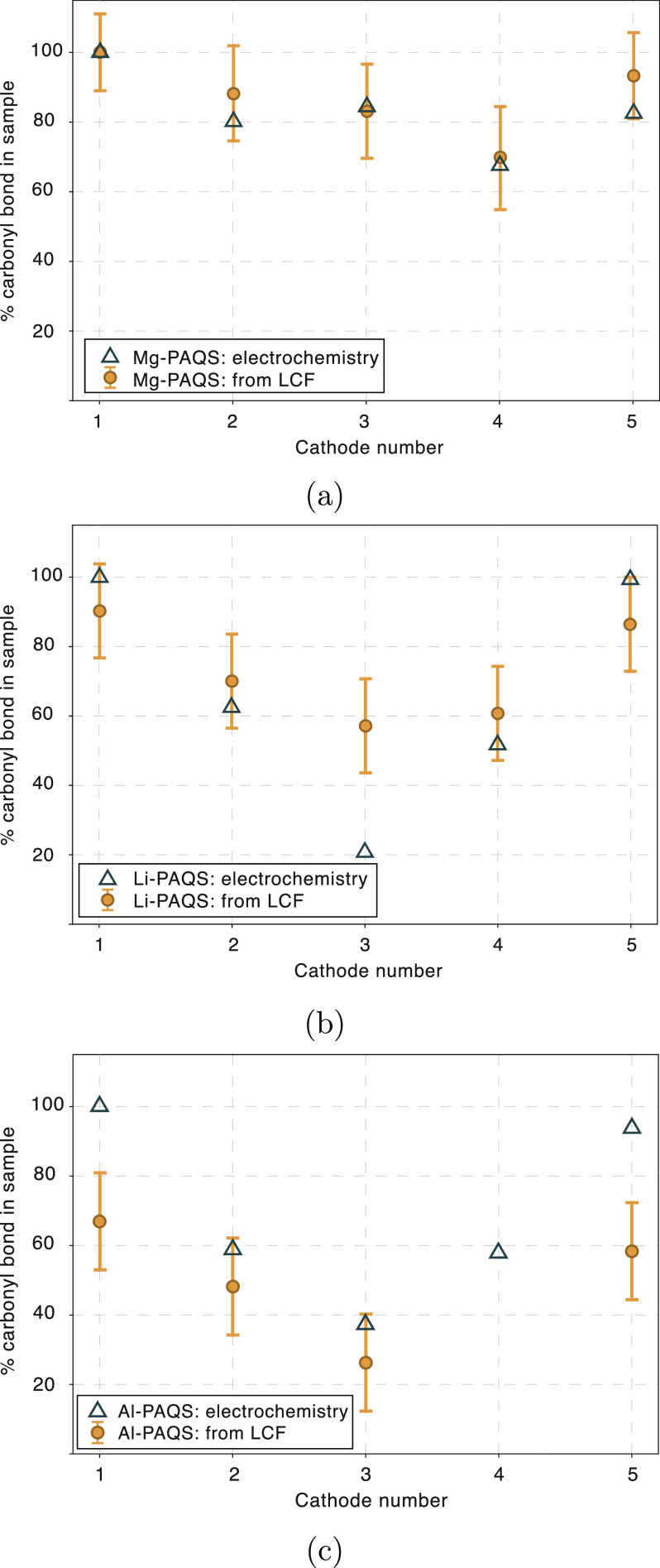
Comparison between relative amounts of carbonyl bond obtained using LCF analysis of measured spectra and by electrochemical characterization for three batteries: (a) Mg-PAQS system, (b) Li-PAQS, and (c) Al-PAQS system. Uncertainty here is a combination of statistical accuracy and error arising from the fact that the initial cathode is not accurately described with the respective standard compound.

We investigated PAQS polymer that has already been used with different multivalent ions (Mg, Al). While PAQS electrochemical activity in Li and Al cell is close to the theoretical one, practical capacity utilization in Mg cell reaches only one-third of the theoretical one. The electrochemical discharge curves for each battery setup are presented in SI S3. Lower electrochemical performance of Mg-PAQS cell is mainly caused by higher polarization of a Mg metal anode, leading to an increased hysteresis of a full cell. For all batteries with PAQS cathode, comparison between the values derived from electrochemical characterization and LCF analysis of ex situ electrodes can be seen in Figure 5. Calculated values from LCF of XRS spectra for Mg-PAQS battery display good agreement with electrochemical ones, while the analysis of Li-PAQS and Al-PAQS battery shows an offset of LCF experimental values from the values obtained from electrochemical characterization, suggesting some systematic error. Overall, results of the LCF analysis of the measured XRS spectra in general follow the trend set by electrochemical characterization of samples, with the amount of carbonyl bond decreasing during discharge and increasing during battery charge.

Since the electrochemical mechanisms behind the redox reactions of PAQ and PAQS polymers are similar and the reduction of oxygen plays the main role in electrochemical conversion for both compounds, a similar rate of carbonyl bond conversion was expected for both systems. This was confirmed by electrochemical characterization of samples. However, by comparing the spectra of PAQ and PAQS cathodes in Figures 4a and S4, it is apparent, that they do not match, and that a large percent of the characteristic resonance yield remains present at the end of the discharge cycle of the PAQS battery, while it almost completely disappears in the PAQ battery. After excluding a systematic error, as explained in SI S4, the difference between the relative amounts of carbonyl bond obtained using LCF and electrochemical characterization was interpreted as a result of an increased presence of stabilized radical species in a fully discharged Li-PAQS cathode. This hypothesis was further investigated by performing additional theoretical calculations and IR ex situ measurements, which are presented in SI S4.1. Analysis of the measured XRS spectra confirmed the reduction of the double carbonyl bond as a mechanism behind the electrochemical activity of metal–organic batteries in both PAQ and PAQS cathodes. The intermediate radical state was also characterized by the theoretical calculations validating the proposed multistep redox reaction shown in Figure 1. The presence of radical species in the fully discharged PAQS cathode was unexpected and the mechanism for their stabilization in different cathode materials has to be investigated further.

## Conclusions

Ex situ X-ray Raman spectroscopy was used to measure the oxygen K-edge XRS spectra of several standard compounds and cathodes from four distinct battery systems (Li-PAQ, Mg-PAQS, Al-PAQS, and Li-PAQS). Individual O K-edge spectra were fitted with a linear combination of reference spectra of the selected standards to track the relative amounts of carbonyl bonds in a battery during charge/discharge cycle. To interpret experimental spectra, ab initio quantum mechanical calculations were performed, and theoretical XAS spectra of reference standards were found to be in a good agreement with the measured ones. Presence of a carbonyl bond was identified by the characteristic resonance at 530 eV, which was confirmed to originate from 1s to π* electronic transition. Relative amounts of carbonyl bond, determined from LCF analysis of the measured spectra, were found to be in a good agreement with electrochemical values, calculated from the measured capacity. For the Li-PAQS battery, analysis of the measured O XRS spectra suggested a lower level of electrochemical conversion at the fully discharged state, when compared to electrochemical data. This anomaly was attributed to an increased presence of stabilized radical species in Li-PAQS battery at the end of the discharge cycle.

Our study confirmed XRS as a viable characterization technique, capable of tracking and quantifying electrochemical conversion of oxygen in the bulk material of the metal–organic batteries. Because of the hard X-ray probe, the method is compatible with the use of in situ battery cells with further potential for implementation of operando characterization of a new generation of metal–organic batteries with a variety of metallic counterions and other similar systems that cannot be accurately probed with XAS.

## Supporting Information Available

S1, Detector image analysis; S2, normalization of the experimental spectra; S3, electrochemical discharge curves; S4, source of the discrepancy between Li-PAQ and Li- PAQS spectra; S4.1, ATR-IR spectra of PAQ and PAQS; and additional references (PDF)
