# Supplementary material for: Characterization of Electrochemical Processes in Metal–Organic Batteries by X-ray Raman Spectroscopy
Source: J Phys Chem C Nanomater Interfaces. 2022 Mar 16;126(12):5435–42. doi: 10.1021/acs.jpcc.1c10622 (PMC8978279; doi:10.1021/acs.jpcc.1c10622)
Supplement: Supplementary file 1 — jp1c10622_si_001.pdf [file jp1c10622_si_001.pdf]

Supporting information for

Characterization of Electrochemical Processes in Metal-Organic Batteries by X-ray Raman Spectroscopy

Ava Rajh,[*,†,‡] Iztok Arčon,[†,¶] Klemen Bučar,[†,‡] Matjaž Žitnik,[†,‡] Marko Petric,[†,§] Alen Vizintin,[‖] Jan Bitenc,[‖] Urban Košir,[⊥] Robert Dominko,[‖] Hlynur Gretarsson,[#,@] Martin Sundermann,[#,@] and Matjaž Kavčič[*,†,‡]

†*Jožef Stefan Institute, Jamova 39, 1000 Ljubljana, Slovenia*

‡*University of Ljubljana, Faculty of Mathematics and Physics, Jadranska ulica 19, 1000 Ljubljana, Slovenia*

¶*University of Nova Gorica, Vipavska 13, SI-5000, Nova Gorica, Slovenia*

§*University of Zagreb, Faculty of Geotechnical Engineering, Hallerova aleja 7, 42000 Varaždin, Croatia*

‖*National Institute of Chemistry, Hajdrihova 19, 1000 Ljubljana, Slovenia*

⊥*University of Ljubljana, Faculty of Chemistry and Chemical Technology, Večna pot 113, 1000 Ljubljana, Slovenia*

#*Deutsches Elektronen-Synchrotron DESY, Notkestraße 85, D-22607 Hamburg, Germany*

@*Max Planck Institute for Chemical Physics of Solids, Nöthnitzer Straße 40, D-01187 Dresden, Germany*

E-mail: ava.rajh@ijs.si; matjaz.kavcic@ijs.si

Phone: +386 30 696 352

S1 Detector image analysis

Image from the 2D Medipix detector is shown in Figure S1a. It was analysed using a python script, which uses the elastic peak to adjust for monochromator drift, evaluates energy resolution of each analyser and converts the incident photon energy into energy loss spectrum. Individual Regions of Interest (ROIs) that contain information about the sample are identified for each analyser with the help of the elastic scan as seen in Figure S1b and the final spectrum is averaged over all 12 analysers. Due to a large penetration depth of X-rays into the bulk material, the image also contains information about the depth profile of the sample. Three distinct regions can be identified in the Figure S1c, each of them corresponding to a different layer with different XRS spectra. In our case it was used to identify and separate signal of the window foil from the signal of the cathode sample. Since the electrode is the only part of the sample that contains oxygen, we have separated the contribution from the electrode by observing, at which depth the oxygen signal appears and disappears. Selecting only the relevant part of the sample increased signal to background ratio and could, in the future, be used to sample chemical composition at different battery depths, providing accurate information about the rate of the redox reaction at different layers in the cell.

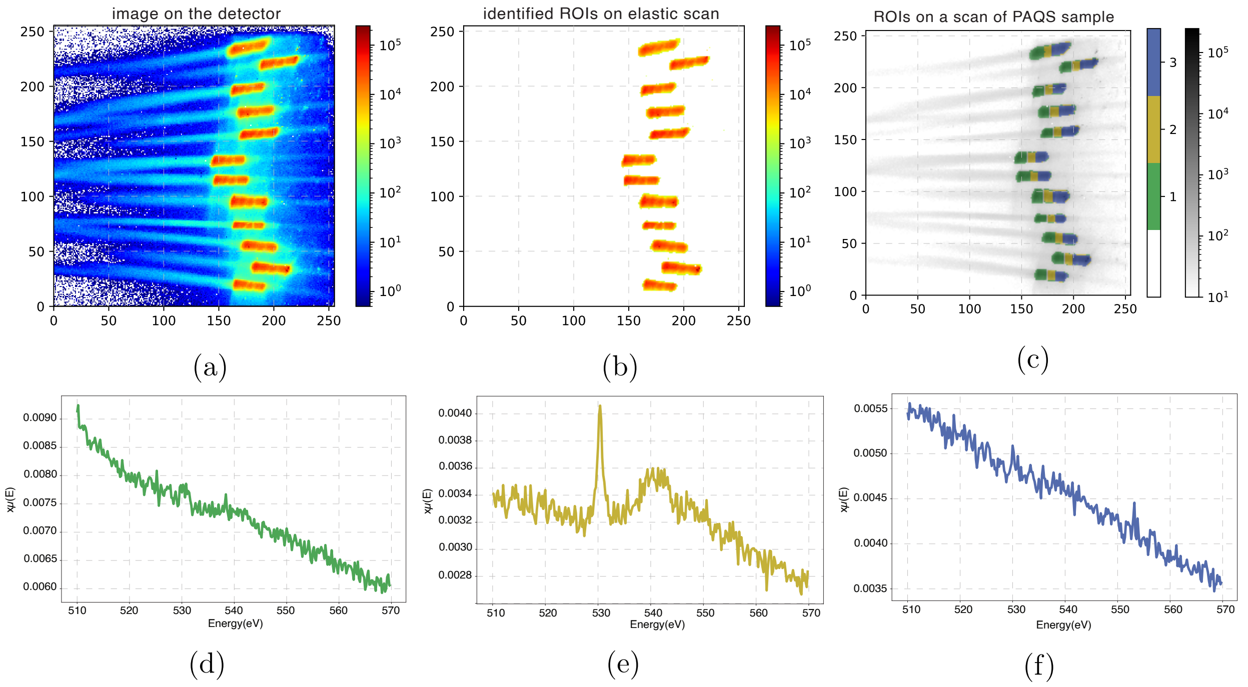

Figure S1: a) 2D image from the detector for one elastic scan of PAQS sample. b) Identified ROIs on the image of the elastic scan. c) Image of one XRS O-K-edge scan of PAQS sample overlayed with a depth profile of the sample. Three distinct regions are identified: window foil (green), sample (yellow), backing material (blue). d) XRS spectrum from window foil, e) XRS spectrum from the sample, f) XRS spectrum from the backing foil.

S2 Normalization of the experimental spectra

Contribution from Compton scattering was subtracted from the measured spectra as a linear background using Demeter Athena software package[1] and data was normalized as shown in Figure S2. Normalization of the experimental spectra was done by choosing an appropriate pre-edge and post-edge regions. For the pre-edge region, we have chosen the range from 510 to 525 eV and for the post-edge region, we have chosen the range from 545 to 559 eV.

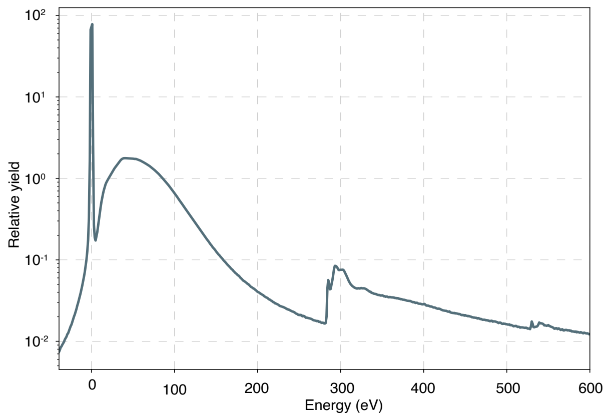

(a)

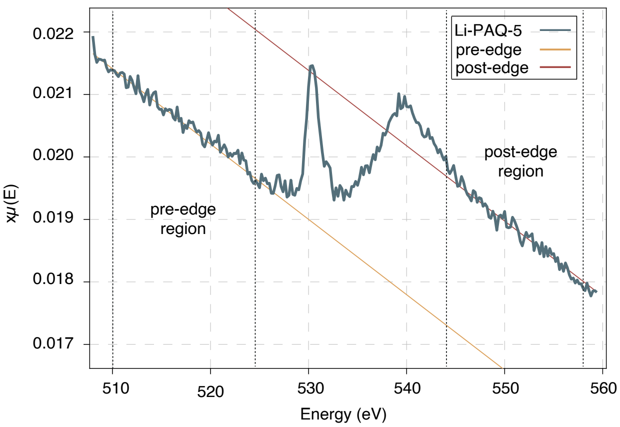

(b)

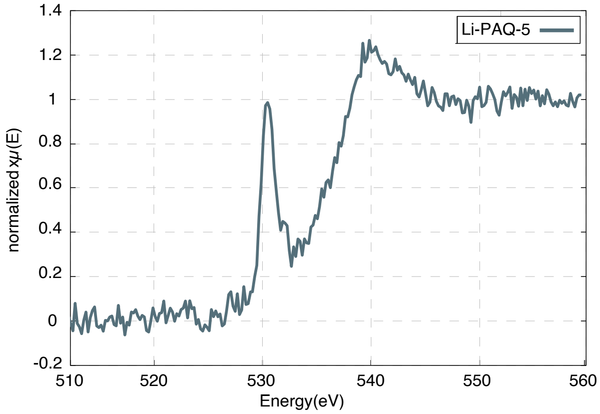

(c)

Figure S2: a) Total XRS spectrum measured on the AQ sample. b) Oxygen K edge XRS spectrum measured *ex situ* on Li-PAQS cathode with pre-edge and post-edge lines used for normalization. c) Normalized XRS spectra with subtracted background.

S3 Electrochemical discharge curves

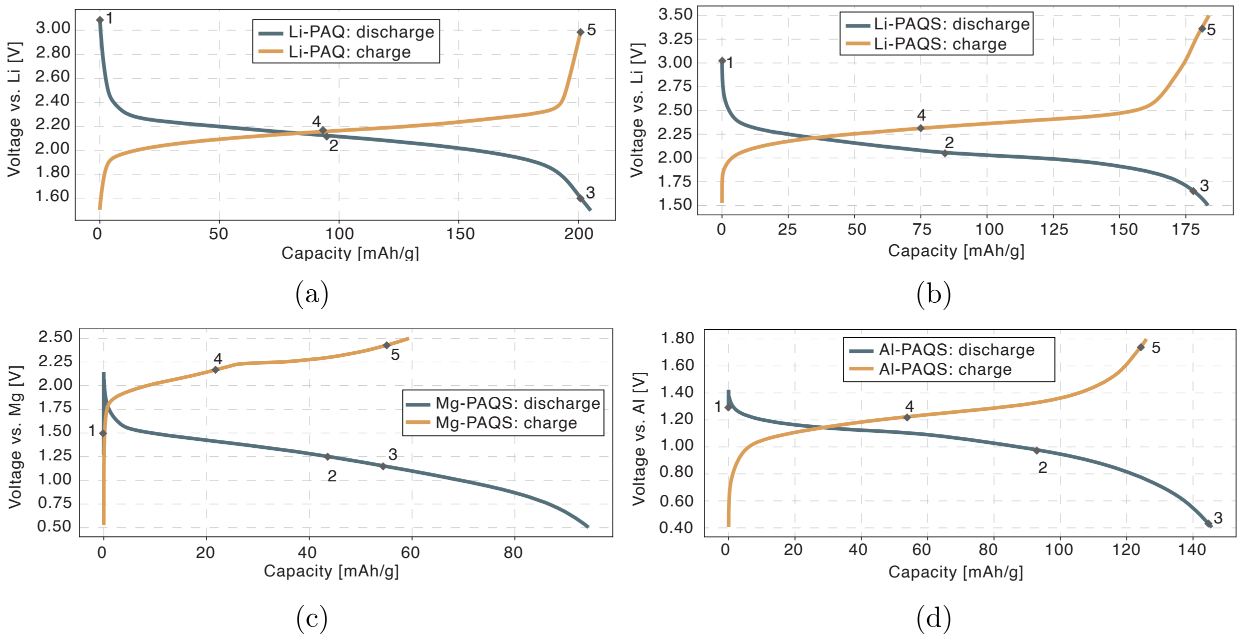

Figure S3: Electrochemical discharge curves of all battery systems with labelled points corresponding to sample cathodes, measured *ex situ*.

The plots in Figure S3 represent a typical electrochemical discharge curves of a battery. Labelled points correspond to the capacity obtained during cycling and only approximately to the specific voltage curve.

S4 Source of the discrepancy between Li-PAQ and Li-PAQS spectra

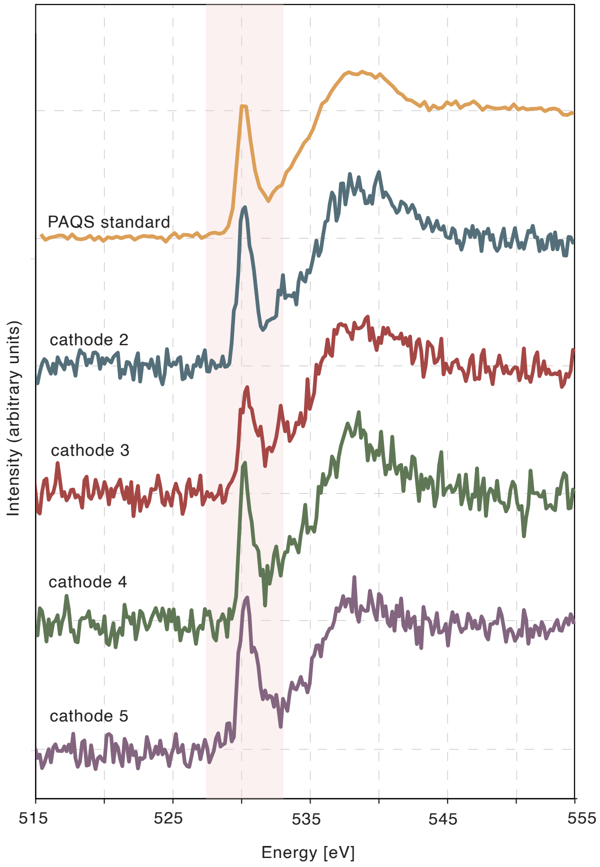

Figure S4: Experimental oxygen K-edge XRS spectra from 5 pre-cycled cathodes measured *ex situ* from Li-PAQS battery.

When comparing the normalized XRS spectra of individual cathodes for Li-PAQS in Figure S4 and for Li-PAQ in Figure 4, along with the results of LCF analysis in Figure 5, a less efficient electrochemical conversion is implied for the Li-PAQS fully discharged cathode. In order to exclude any problems with the specific set of samples, additional independent measurements were performed on a second set of Li-PAQS cathodes, but a similar deviation from the expected electrochemical values was observed. This confirmed that the anomaly is

a result of some unknown systematic effect that influences the shape of XRS spectra, but that does not change the overall cell electrochemistry.

By comparing the theoretical XAS spectra of PAQ and PAQS molecules in Figure S5a, it was confirmed that the addition of sulphur atoms has no influence on the final spectrum and an investigation into other factors that could influence the shape of XRS spectra was performed. Since radical molecule PAQS* plays an important role in the electrochemical process, a possible reason for the difference could be the presence of stabilized intermediate radical species in a cathode sample. In LCF analysis, two reference spectra were used, representing initial and final stages in a battery cycle (PAQS and Li_2AQ), so the presence of a third distinct intermediate state could influence the shape of the measured spectra and consequently alter the result of LCF analysis. To confirm this hypothesis, theoretical XAS spectra of radical species LiAQ* and LiAQS* were calculated. Their comparison with theoretical spectra of a fully reduced state can be seen in Figure S5b and shows that the intermediate radical state retains some of the pre-edge resonance, characteristic of the carbonyl bond. Within LCF analysis, the presence of radical species could be interpreted as an increased presence of the initial PAQS state in the fully discharged system. The analysis of the measured spectra therefore implies, that radical species, which form during the redox reaction, are stabilized in the Li-PAQS cathode, leading to an increased amount of radicals in samples towards the end of the discharge, compared to the amount in the Li-PAQ cathode.

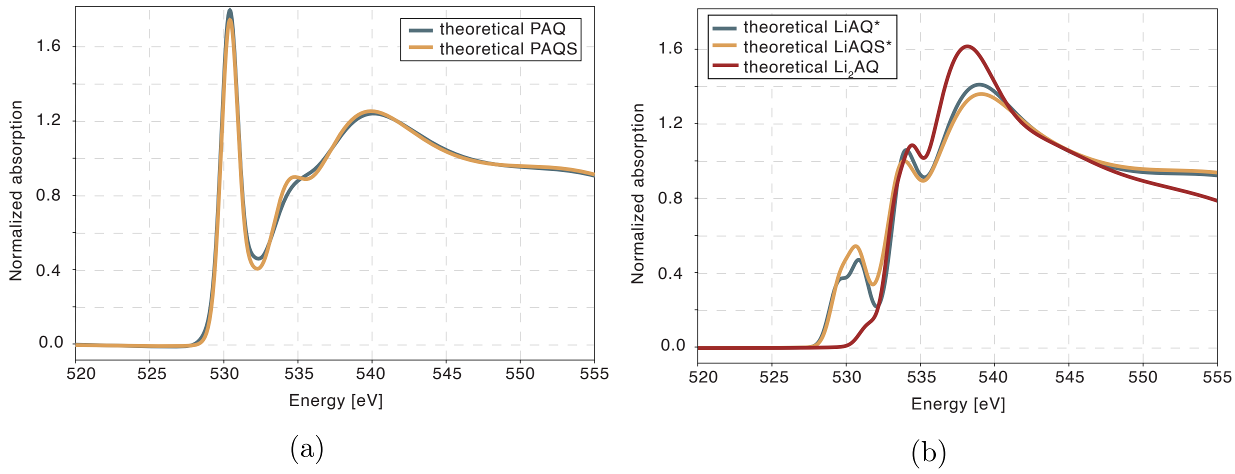

Figure S5: a) Theoretical oxygen XAS spectra for PAQ and PAQS molecules; b) Theoretical oxygen XRS spectra for radical species LiAQ* and LiAQS*, compared to XAS spectra of Li2AQ molecule.

S4.1 ATR-IR spectra of PAQ and PAQS

To further investigate the difference between Li-PAQ and Li-PAQS batteries, ATR-IR spectra of standard samples and both fully discharged cathodes were recorded, as described in[2,3]. Comparison of the reference spectra AQ, representing the beginning stage of a battery cycle and Li_2AQ, representing a fully discharged state of a battery is seen in Figure S6. DFT was used to assign vibrational modes to experimental vibrational bands. In the Li_2AQ spectrum, a decrease in the intensity of C=O band at 1677 cm^{-1} is observed along with an increase in the intensity of the C-O^{-} at 1374 cm^{-1},

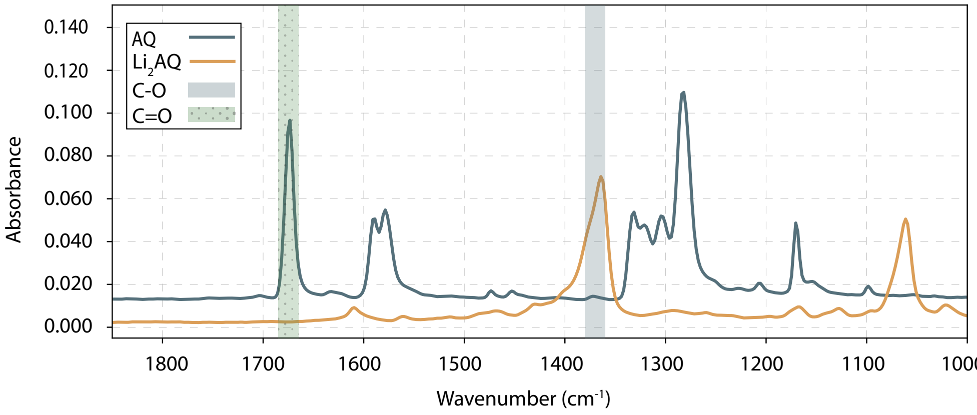

Figure S6: ATR-IR characterization of standards samples AQ and Li_2AQ.

When comparing the spectra of fully discharged cathodes Li-PAQ and Li-PAQS measured *ex situ* pictured in Figure S7, C=O band is missing in both accompanied by a prominent band representing a C-O^{-1} bond, confirming that the cathodes are at the end of the discharge cycle. Additional bands were identified with the help of DFT at 1500-1490 and 1400-1390 cm^{-1} belonging to a radical state. While there is not much difference between the two spectra at the 1400-1390 cm^{-1} band, the one at 1500-1490 cm^{-1} is clearly more intense for the Li-PAQS cathode implying a larger presence of radical species.

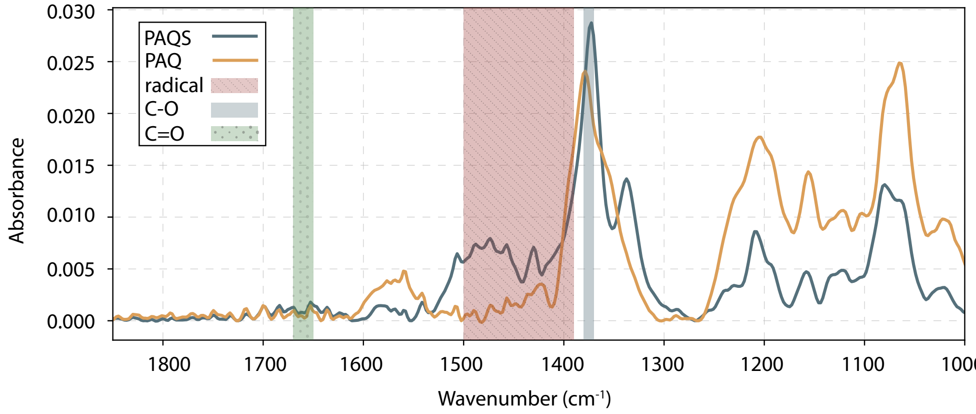

Figure S7: ATR-IR characterization of fully discharged Li-PAQ and Li-PAQS cathodes measured *ex situ*.

References

(1) Ravel, B.; Newville, M. *ATHENA*, *ARTEMIS*, *HEPHAESTUS*: Data Analysis for X-ray Absorption Spectroscopy Using *IFEFFIT*. *Journal of Synchrotron Radiation* **2005**, *12*, 537–541.

(2) Vizintin, A.; Bitenc, J.; Kopač Lautar, A.; Pirnat, K.; Grdadolnik, J.; Stare, J.; Randon-Vitanova, A.; Dominko, R. Probing Electrochemical Reactions in Organic Cathode Materials via In Operando Infrared Spectroscopy. *Nature communications* **2018**, *9*, 661.

(3) Bitenc, J.; Vizintin, A.; Grdadolnik, J.; Dominko, R. Tracking Electrochemical Reactions Inside Organic Electrodes by Operando IR Spectroscopy. *Energy Storage Materials* **2019**, *21*, 347–353.
